# Work ability and physical fitness among aging workers: the Finnish Retirement and Aging Study

**DOI:** 10.1007/s10433-022-00714-1

**Published:** 2022-06-22

**Authors:** Kristin Suorsa, Ville-Mikko Mattila, Tuija Leskinen, Olli J. Heinonen, Jaana Pentti, Jussi Vahtera, Sari Stenholm

**Affiliations:** 1 Department of Public Health, University of Turku and Turku University Hospital, Turku, Finland; 2 Centre for Population Health Research, University of Turku & Turku University Hospital, Turku, Finland; 3 Paavo Nurmi Centre and Unit for Health and Physical Activity, University of Turku, Turku, Finland; 4 Clinicum, Faculty of Medicine, University of Helsinki, Helsinki, Finland

**Keywords:** Fitness testing, Muscle strength, Cardiorespiratory fitness, Capacity for work

## Abstract

**Background:**

With advancing age, physical capacity gradually decreases which may lead to decreased work ability, if the physical work requirements remain the same. Examination of the importance of physical fitness for work ability among aging workers will help to find potential strategies to promote work ability in old age. The aim of this study was to investigate the association between physical fitness and work ability among aging workers.

**Methods:**

Aging workers (*n* = 288, mean age 62.5, 83% women) from the Finnish Retirement and Aging study underwent cardiorespiratory, muscular fitness and functional testing. Work ability was inquired on a scale 0–10 from poor to excellent. Association between physical fitness indicators and work ability was examined using ordinary least squares regression, taking into account age, gender, occupational status, heavy physical work, body mass index and accelerometer-measured daily total physical activity.

**Results:**

VO2peak, modified push-up test and maximal walking speed were positively associated with work ability (*β* = 0.51, 95% confidence interval (CI) 0.29–0.74, *β* = 0.46, 95% CI 0.26–0.66 and *β* = 0.23, 95% CI 0.07–0.39, respectively), while chair rise test time was inversely associated with work ability (*β* = −0.23, 95% CI −0.39–−0.06). No associations were found between hand grip strength or sit-up test and work ability.

**Conclusions:**

Cardiorespiratory fitness, upper body strength, and lower extremity function were positively associated with work ability. Good physical fitness may help to maintain work ability among aging workers.

**Supplementary Information:**

The online version contains supplementary material available at 10.1007/s10433-022-00714-1.

## Introduction

With population aging and increase in retirement ages, the proportion of aging workers of the working force is increasing rapidly in Western countries. Aging workers experience age-related gradual decline in their cardiovascular and musculoskeletal fitness, which may lead to imbalance between worker’s physical capacity and work’s demands, if the physical work requirements remain the same (Ilmarinen et al. 1997; Kenny et al. 2008; Savinainen et al. 2004). Work ability is one way of evaluating the balance between worker’s physical capacity and work’s demands, and poor work ability has shown to predict functional capacity as indicated, e.g., by sickness absences (Kinnunen and Nätti 2018; Reeuwijk et al. 2015), early exit from the workforce (Salonen et al. 2003) and disability retirement (Bethge et al. 2021; Jääskeläinen et al. 2016; Kinnunen and Nätti 2018). Thus, good work ability can be considered as a key factor in promoting work life participation among aging workers. Keeping aging workers in the working force as long as possible has been one important policy in aging societies to maintain health and social systems, production levels and sustainability during the last decades (D’Addio et al. 2010).

Examination of factors associated with work ability among aging workers could potentially help in the planning of interventions to promote work ability in old age. Muscular and cardiorespiratory fitness are important traits in maintaining good functional capacity as people get older (Foldvari et al. 2000; Reid and Fielding 2012). The most comprehensive examinations of the association between both muscular and cardiorespiratory fitness and perceived work ability have been conducted among municipal workers in the 1990’s (Nygård et al. 1991), female home care workers in the early 2000’s (Pohjonen 2001), and male construction and manufacturing workers in years 2007–2010 (Smolander et al. 2010; Sörensen et al. 2007). In these studies, muscular strength and endurance of upper body, trunk and lower body muscles were found to associate positively with work ability (Nygård et al. 1991; Pohjonen, 2001; Smolander et al. 2010), but findings were somewhat conflicting for cardiorespiratory fitness (Nygård et al. 1991; Pohjonen 2001; Sörensen et al. 2007). During the last 10 years, studies have focused on the associations between muscular strength (upper body, trunk) and work ability among middle-aged employees mostly from manual occupations, and modest associations have been found (Boschman et al. 2017; Edlund et al. 2012; Ezzatvar et al. 2021). However, work life has changed during the last decades in the developed countries, as many physical work tasks have become less physically strenuous due to technological developments, and sedentary office work has become more common (Ng and Popkin 2012). Some evidence suggests that physical fitness have become relevant not only in physical jobs, but also in mentally demanding jobs, as those with better physical fitness have shown better performance in cognitive tests (Dalager et al. 2021). Thus, the association between physical fitness and work ability needs to be re-evaluated in the current work life and among aging workers.

The aim of this study was to examine how physical fitness, both cardiorespiratory and muscular, and physical functioning are associated with perceived work ability among aging public sector workers in Finland.

## Methods

### Study population

Finnish Retirement and Aging Study (FIREA) is an ongoing longitudinal cohort study of aging public sector workers in Finland established in 2013, described previously in detail (Leskinen et al. 2018). Those Finnish speaking FIREA survey cohort study participants who had responded to the survey at least once, whose estimated retirement date was between 2017 and 2019, who lived in the Southwest Finland and were still working, were invited to participate in this clinical sub study (*n* = 773). Of them, 290 consented and participated in the clinical sub study.

The clinical sub study participants answered to the baseline survey between September 2014 and November 2017. On average 4 months after responding to the baseline survey, the study participants attended a clinical visit and participated in accelerometer measurements. Within 1 month from the clinical visit, the participants took part in a physical fitness test battery. Two participants were excluded from the current study because they did not answer the question regarding work ability, resulting in an analytic sample of 288 study participants.

The FIREA study was conducted in accordance with the Helsinki declaration and was approved by the Ethics Committee of Hospital District of Southwest Finland. All participants provided informed consent.

### Assessment of work ability

Work ability was inquired at the baseline survey with a question: “Assume that your work ability at its best has a value of 10 points and 0 would mean that you could not work at all. What score would you give to your current work ability?” This score is called work ability score (WAS) and it is the first item of multi-item work ability index (WAI) (Ilmarinen et al. 1997). WAS ranges from 0 (completely unable to work) to 10 (work ability at its best). WAS is a reasonable alternative to the seven-item WAI as a prognostic tool to identify risk of disability pension (Fassi et al. 2013; Jääskeläinen et al. 2016; Lundin et al. 2017). We used WAS both as a continuous and as categorical measure with four groups: poor (0–5 points), moderate (6,7), good (8,9), excellent (10) (Ilmarinen et al. 1997).

### Assessment of physical fitness

After the baseline survey, participants took part in a clinical visit, which was conducted by a study nurse and included following physical performance tests: hand grip strength, chair rise and maximal walking speed tests.

Hand grip strength was used to examine upper body strength and measured in Newtons (N) using a handheld dynamometer (Jamar) (Suni et al. 2014; Suni et al. 1998). The measurement was taken with the dominant hand with the participant seated, elbow flexed at a 110° angle, wrist in a neutral position, and the interphalangeal joint of the index finger at a 90° angle. The participant was instructed to squeeze the handle with maximal effort for 3–5 s and standard encouragement for maximal performance was given. The measurement was repeated after a 30-s pause for recovery. If the two results differed more than 10%, a third attempt was made. The best result was used in the analyses. Two participants could not perform the test due to osteoarthritis and one participant due to recent operation.

Chair rise test was used to assess lower extremity function (Guralnik et al. 1995). Participants were asked to stand up and sit down as quickly as possible 10 times with their hands folded across their chest. Time (in seconds) to complete the test was recorded. Two participants did not want to participate in the chair rise test and one participant were unable to perform the test due to back pain.

Maximal walking speed was also used to evaluate lower-extremity function and measured over a distance of 4-m using a stopwatch (Guralnik et al. 1995). Participants were instructed to “walk to the end of the course as fast as you can,” starting from a standstill. Time (in seconds) was recorded and speed (m/s) was calculated. All participants performed the test.

Within 1 month from the clinical visit, participants attended a physical fitness test battery conducted by an experienced exercise physiologist, which included following physical fitness tests: modified push-up test, sit-up test and indirect submaximal bicycle ergometer test. Twenty-nine participants were unwilling to participate in the physical fitness test battery.

The modified push-up test was used to examine upper-body strength and stability (Suni et al. 1998). Participants were asked to perform as many pushups as possible during 40 s and number of repetitions were calculated. If participant was unable to successfully perform at least one push-up, then value 0 was given. Twenty-one participants did not perform the test due to musculoskeletal pain in the upper body or back and 19 had contraindications (arrhythmia, coronary artery disease, myocardial infarction or other cardiovascular disease).

The sit-up test was used to evaluate dynamic muscular endurance of the trunk flexor (Taulaniemi and Kankaanpää 2015). Participants were asked to perform five repetitions in each of the three performance levels with increasing difficulty (Suni 2009), and number of successful repetitions were calculated. Two participants were unwilling to perform the test and 19 had contraindications (arrhythmia, coronary artery disease, myocardial infarction or other cardiovascular disease).

After the individually performed muscular fitness tests, cardiorespiratory fitness was measured with indirect submaximal bicycle ergometer (Ergoselect 100 K (Ergoline, Germany)) test following the procedure reported previously (American College of Sports Medicine, 2014; Stenholm et al. 2021). Shortly, the test included a 5-min warm-up period and 12-min test, consisting of three graded workloads until 85–90% of the age-predicted maximal heartrate (210–0.65*age) or the rating of perceived exertion over 15 was achieved. Maximal working capacity was extrapolated by using workloads and heartrates collected during the test and it was then converted into peak oxygen consumption (VO2peak) (ml/kg/min) by using formula (11.02*watts)/body weight + 7. One participant was unwilling to participate in the test and 19 participants were unable to perform the test due to contraindications (arrhythmia, coronary artery disease, myocardial infarction or other cardiovascular disease).

### Assessment of participant characteristics

Age (calculated from date of birth), occupational status and sex of the study participants were obtained from the register of municipal sector’s pension insurance institute in Finland (Keva). Occupational status was classified by International Standard Classification of Occupations (ISCO) and was categorized in three groups: upper-grade non-manual (e.g., physicians, teachers, administrators), lower-grade non-manual (e.g., registered nurses, secretaries, practical nurses, cooks) and manual or service work (e.g., maintenance workers, cleaners) (Statistics of Finland 2010). Heavy physical work (no vs. yes) was assessed by using validated gender-specific job exposure matrix for physical exposures (Solovieva et al. 2012).

Body mass index was calculated based on height and weight which the study nurse measured during the clinical study visit. Daily total wake time physical activity was assessed with accelerometer measurements which were conducted close to the clinical study visit by mailing triaxial ActiGraph wActiSleep-BT accelerometers (ActiGraph, Pensacola, FL, US) with instructions to the study participants. Participants wore the accelerometer on the non-dominant wrist on average 9 days (range 3–13, including 4.5 workdays and 4.2 days off) at all times, including water-based activities (Suorsa et al. 2020). Triaxial acceleration data were processed to vector magnitude (VM) counts per minute (CPM), daily mean CPM was calculated and averaged across the whole measurement period following the principles reported elsewhere (Pulakka et al. 2019).

Other participant characteristics were derived from the baseline survey. Self-reported leisure time physical activity was assessed with a question on average weekly duration and intensity of leisure and commuting physical activity during the past year, and metabolic equivalent (MET) hours per week were calculated. Smoking was categorized as non-smokers or former smokers and current smokers. Chronic diseases diagnosed by a physician (angina pectoris, myocardial infarction, cerebrovascular disease, cancer, osteoarthritis, rheumatoid arthritis, diabetes and asthma) were categorized as having no disease, or one or more diseases. Mobility limitations were assessed with the validated RAND-36 Health Survey (identical with the Short Form SF-36) (Aalto et al. 1999; Hays et al. 1993) and for the current study we used information on mobility limitation based on the question of difficulties in walking 2 km (no/some or marked difficulties).

### Statistical analysis

Study population characteristics by work ability categories are reported as mean and standard deviation for continuous variables and proportions for categorical variables.

Association between physical fitness indicators and work ability was examined with ordinary least squares (OLS) regression and results are shown as beta coefficients and their 95% confidence intervals (CI). Physical fitness indicators were used as standardized values (divided by standard deviation (SD)) in the analyses. Analyses were first adjusted for age, sex and occupational status (Model 1). In the second set of analyses, model was additionally adjusted for heavy physical work, body mass index and daily total physical activity (Model 2). To examine whether association between physical fitness and work ability depended on occupation, an interaction term physical fitness indicator*occupational status was added to the OLS regression Model 1, where occupational status was used as a dichotomous variable (manual vs. non-manual workers). Furthermore, to illustrate association between physical fitness and work ability, ANCOVA was used to examine categorical work ability measure and each physical fitness indicator. The results are presented as means and their 95% CIs for each work ability group, adjusting for age, sex and occupational status.

To examine selection bias, work ability and other participant characteristics were compared between the current study population and the FIREA survey cohort participants who belonged only to the survey population and were still working at the time of the baseline survey (*n* = 4670). Furthermore, the study population with physical performance tests (*n* = 288) were compared with the study population with physical fitness tests (*n* = 239). Differences between the groups were examined using Chi squared test for categorical variables and ANOVA for continuous variables.

Statistical analysis was performed using statistical software SAS version 9.4 (SAS Institute Inc.).

## Results

Characteristics of the study population are shown in Table 1. Mean age of the study population was 62.5 (SD 1.0) and 83% of all participants were women. Of the participants 6% reported poor, 24% moderate, 59% good and 11% excellent work ability. There were no significant differences in baseline characteristics across work ability groups (Table 1).

**Table 1:** Characteristics of the study population

Characteristics	All	Work ability category				
		Poor	Moderate	Good	Excellent	*p* value
*N* (%)	288	16 (6)	69 (24)	171 (59)	32 (11)	
Age, mean (SD) years	62.5 (1.0)	62.2 (1.1)	62.4 (1.1)	62.6 (1.0)	62.5 (1.1)	0.21
*Gender*						0.59
Women, *n* (%)	239 (83)	14 (88)	57 (83)	139 (81)	29 (91)	
Men, *n* (%)	49 (17)	2 (13)	12 (17)	32 (19)	3 (9)	
*Occupational status, n *(%)						0.10
Upper-grade non-manual	102 (35)	3 (19)	19 (28)	67 (39)	13 (41)	
Lower-grade non-manual	97 (34)	4 (25)	28 (41)	52 (30)	13 (41)	
Manual/service	89 (31)	9 (56)	22 (32)	52 (30)	6 (19)	
Heavy physical work, *n* (%)	49 (17)	5 (31)	12 (17)	29 (17)	3 (9)	0.30
Body mass index, kg/m^2^, mean (SD)	26.3 (4.6)	27.7 (4.6)	25.9 (3.9)	26.3 (4.6)	26.1 (5.7)	0.61
Daily physical activity, VM CPM^A,^ mean (SD)	2470 (569)	2595 (670)	2402 (442)	2468 (606)	2565 (554)	0.60

^A^Vector magnitude (VM) counts per minute (CPM)

**Fig. 1:**
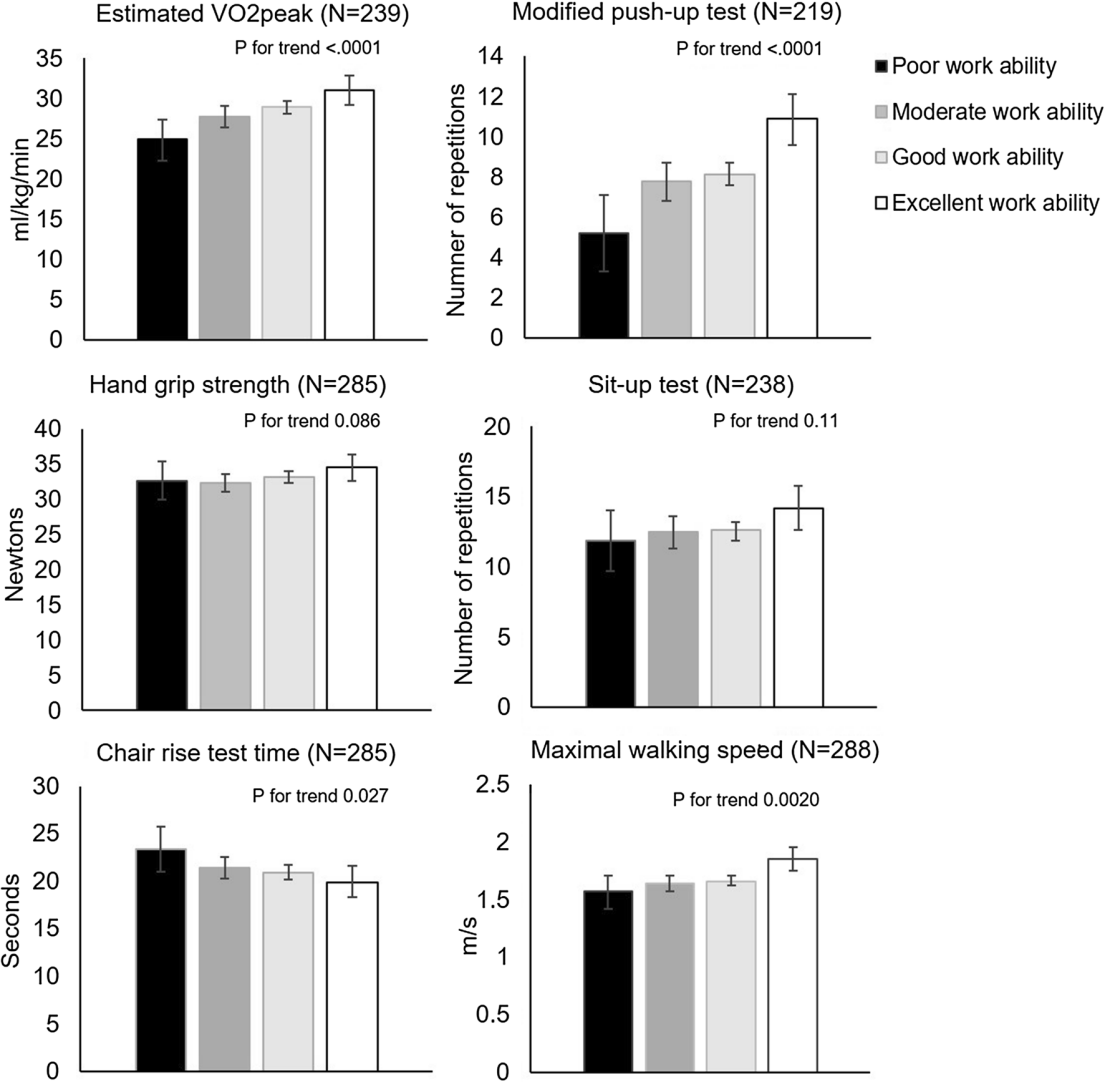
Physical fitness measures among poor, moderate, good and excellent work ability groups expressed as means and their 95% confidence intervals, adjusted for age, gender and occupational status

Table 2 and Fig. 1 present the association between physical fitness and work ability. Estimated VO2peak, modified push-up test score and maximal walking speed were positively associated with work ability (in the fully adjusted model, *β* = 0.51, 95% CI 0.29–0.74, *β* = 0.46, 95% CI 0.26–0.66 and *β* = 0.23, 95% CI 0.07–0.39, respectively). Moreover, chair rise test time and work ability were inversely associated (in the fully adjusted model *β* = −0.23, 95% CI −0.39–−0.06) (Table 2). Compared to those who evaluated their work ability to be poor, the excellent work ability group had higher estimated VO2peak (mean 24.9 ml/kg/min, 95% CI 22.3–27.4 vs. 31.0 ml/kg/min, 95% CI 29.2–32.8), repetitions in modified push-up test (mean 5.2, 95% CI 3.3–7.1 vs. 10.9, 95% CI 9.6–12.1) and maximal walking speed (mean 1.6 m/s, 95% CI 1.4–1.7 vs. 1.8 m/s, 95% CI 1.7–2.0) (Fig. 1). Furthermore, chair rise test time was shorter among the excellent work ability group compared with the poor work ability group (mean 19.9 s, 95% CI 18.3–21.6 vs. 23.4 s, 95% CI 21.0–25.7) (Fig. 1). There was no association between work ability and hand grip strength or sit-up test.

**Table 2:** Association between physical fitness and work ability

Physical fitness indicator	Model 1^A^				Model 2^B^			
	*β*^C^	95% CI		*p* value	*β*^C^	95% CI		*p* value
Estimated VO2peak (ml/kg/min)	0.34	0.16	0.51	< .001	0.51	0.29	0.74	< .001
Modified push-up test (number of repetitions)	0.42	0.24	0.60	< .001	0.46	0.26	0.66	< .001
Hand grip strength (N)	0.12	− 0.13	0.36	0.35	0.11	− 0.14	0.36	0.39
Sit-up test (number of repetitions)	0.08	− 0.09	0.25	0.36	0.10	− 0.10	0.29	0.32
Chair rise test time (s)	− 0.23	− 0.38	− 0.07	0.004	− 0.23	− 0.39	− 0.06	0.007
Maximal walking speed (m/s)	0.24	0.09	0.40	0.002	0.23	0.07	0.39	0.005

^A^Adjusted for age, gender and occupational status

^B^Additionally adjusted for heavy physical work, body mass index and daily total physical activity

^C^ *β* indicates change in work ability for every one-unit SD (standard deviation) change of physical fitness indicator

To examine the role of occupational status on the association between physical fitness indicators and work ability, the interaction term physical fitness indicator*occupational status was added to the Model 1. The results are presented in Supplementary Table 1. No significant interactions were found for any of the physical fitness indicators (*p* values between 0.40 and 0.97).

Table 3 shows the additional analyses regarding selection into the study population. Comparison between the current study population and the FIREA survey cohort showed that the current study population had slightly higher work ability compared to the survey-only study population still working at the time of the baseline survey (8.0 vs. 7.8) (Table 3). Moreover, the current study population had lower BMI, was more physically active and less likely smokers, and had less likely mobility limitations compared with the survey-only study participants. There were no differences in terms of age, gender, occupational status, heavy physical work or chronic diseases.

**Table 3:** Comparison between the study population and the FIREA survey cohort participants

Characteristic	FIREA survey cohort^A^	Study population	Study population which participated in the physical fitness test battery	*p* value FIREA survey cohort vs. study population	*p* value Study population which participated in the physical fitness test battery vs. study population
*N*	4670	288	239	–	–
Age, mean (SD)	62.6 (1.1)	62.5 (1.0)	62.4 (1.0)	0.14	0.0090
Women, %	82	83	82	0.59	0.33
Occupational status, %				0.087	0.54
Upper-grade non-manual	32	35	37	–	–
Lower-grade non-manual	30	34	33	–	–
Manual/service	37	31	30	–	–
Work ability, mean (SD)	7.8 (1.6)	8.0 (1.4)	8.1 (1.3)	0.022	0.079
Heavy physical work, %	15	17	16	0.45	0.27
Body mass index, kg/m^2^, mean (SD)^B^	26.8 (4.5)	25.9 (4.0)	25.5 (3.6)	0.0022	0.0001
Self-reported physical activity, MET-hours/week, mean (SD)	23 (20)	29 (22)	30 (22)	< .0001	0.19
Current smoking, %	11	5	5	0.0018	0.65
Chronic diseases, %	63	65	63	0.56	0.21
Mobility limitation, %	15	9	6	0.0024	0.0014
Maximal walking speed (m/s), mean (SD)	NA	1.7 (0.3)	1.7 (0.3)	NA	0.11
Hand grip strength (*N*), mean (SD)	NA	33.1 (8.5)	33.3 (8.8)	NA	0.27
Chair rise test time (*s*), mean (SD)	NA	21.1 (4.7)	20.5 (3.9)	NA	< .0001

^A^Those who belonged only to the survey cohort and were still working at the time of the baseline survey

^B^Based on self-reported height and weight from the baseline survey

Comparison of the study participants with physical performance tests (*n* = 288) and study participants with the physical fitness tests (*n* = 239) showed that the participants did not differ in terms of gender, occupational status, work ability, heavy physical work, self-reported physical activity, smoking or chronic diseases. However, those with physical fitness tests were slightly younger (*p* = 0.0090), had lower BMI (*p* = 0.0001), their mobility was less limited (*p* = 0.0014) and they performed better in the chair rise test (*p* < 0.0001) compared to those with physical performance tests (Table 3).

### Discussion

Work ability and cardiorespiratory fitness, upper body strength and lower extremity function were positively associated among aging workers. Associations were found even after controlling for heavy physical work and accelerometer-measured daily total physical activity. Hand grip strength or sit-up test were not associated with work ability. Our findings can be utilized in planning of occupational health interventions to promote work ability among aging workers working in various occupations.

The results of the current study extend and update previous knowledge on the association between work ability and physical fitness to aging public sector workers aged over 60 years and in the last years of their working career. Of the muscular fitness tests, we found only modified push-up test to associate with work ability, whereas a previous study among Finnish middle-aged municipal workers from the 90’s reported associations between hand grip and trunk strength and work ability (Nygård et al. 1991). Other studies, conducted among middle-aged employees from specific manual occupational groups, found that not only upper body and trunk strength, but also lower body strength were positively associated with work ability (Pohjonen 2001; Smolander et al. 2010). One explanation for these conflicting findings between our study and the earlier studies may be that work life has become less strenuous during the last three decades in the developed countries due to technological developments (Boschman et al. 2017; Ng and Popkin 2012) and muscular work is no longer needed in the majority of the occupations. However, muscular fitness seems to explain work ability especially in physically strenuous occupations, probably because muscular peak loads are often achieved in lifting and carrying tasks (Boschman et al. 2017; Edlund et al. 2012; Pohjonen 2001; Smolander et al. 2010). At this stage, we did not observe the association to differ between manual and non-manual occupations. Another explanation may be that the hand grip strength and sit-up tests measure relatively specific physical fitness, isometric strength of the forearm muscles and muscle endurance of the trunk flexor muscles; which are mainly needed in physically strenuous work, such as lifting and carrying heavy loads. Physically strenuous work and heavy lifting were rare in our non-manual worker-dominated study population (17% and 12%, respectively). The rest of the tests were more comprehensive, challenging several large muscle groups, balance and coordination, which may partly explain the stronger observed associations with work ability. Moreover, variation in the muscular fitness test results seemed somewhat low between the groups, except for the modified push-up test, which may partly explain why the push-up test was the only muscular test associated with work ability in this age group.

Interestingly, we observed association with cardiorespiratory fitness and work ability among aging public sector workers. Previous studies have reported somewhat mixed findings, as no association was found between estimated VO2peak and work ability among municipal workers (Nygård et al. 1991), whereas in another study among manual workers, estimated VO2peak was associated with work ability, but directly measured VO2peak was not (Sörensen et al. 2007). These somewhat mixed findings could be explained by the fact that physically strenuous job tasks stress mainly musculoskeletal system and less cardiorespiratory system (Nygård et al. 1991). Moreover, cardiorespiratory fitness generally associates positively with self-rated health (Olsson et al. 2018), and self-rated health has been observed to predict work ability so that those with good self-rated health sustain their work ability better compared to those with poor self-rated health (Håkansson et al. 2020). Good cardiorespiratory fitness has also been linked to attenuated response to work-related psychosocial stress (Mucke et al. 2018; Schilling et al. 2020) and better recovery from work, based on heart rate variability measures (Teisala et al. 2014).

The time period after the age of 50 has shown to be critical time point for maintaining work ability (Ilmarinen et al. 1997), mainly because of the age-related decline in physical capacity and negative changes in the health status (Ilmarinen et al. 1997; Kenny et al. 2008). Also, decrease in work ability challenges ones’ general well-being and ability to work until the official retirement age (Bethge et al. 2021; Kinnunen and Nätti 2018). Despite of these challenges faced by aging workers, to maintain the health and social systems and production levels, many countries have implemented strategies to close off early retirement pathways and to encourage aging workers to extend their working careers (D’Addio et al. 2010). The results of this study suggest that promoting cardiorespiratory fitness, upper body strength and lower extremity function may be possible strategies for occupational health interventions to support aging workers’ work ability during the last part of their working careers. Another strategy may be modifying work’s physical requirements to correspond to aging worker’s physical fitness so that there is a balance between aging worker’s work ability and work’s physical requirements.

Strengths of this study include the wide range of fitness and functional tests among aging workers, which enabled us to examine both muscular and cardiorespiratory fitness as correlates of work ability. As a unique feature of this study, analyses were adjusted for accelerometer-measured daily total physical activity, which is not subject to recall or information bias like (the most often used) self-reported physical activity measures (Chastin et al. 2014).

Our study naturally has some limitations. The cross-sectional study design is the main limitation and therefore causal conclusions cannot be made. Other limitations include a small sample size and health-related selection as all participants had better self-reported work ability and they were aging workers from mainly non-manual occupations, having healthy lifestyles and no marked mobility limitations when compared to the FIREA survey population. However, since we found association between work ability and physical fitness even in this relatively healthy study population, the observed associations could be stronger in a more representative study populations. Moreover, there was on average 5-month delay between the baseline survey and the fitness test battery. However, this delay unlikely affected the observed associations between work ability and physical fitness measures because we have observed in a FIREA subpopulation that self-reported work ability does not change in repeat surveys with 3–4 month intervals (data not shown). As the majority of the subjects were female having good and excellent work ability, the generalization of the results to men and those with poor work ability should be done with caution. As one limitation, not all study participants took part in the more demanding fitness test such as the VO2peak test. Although those who participated in the VO2peak test did not differ in terms of work ability or health status from the rest of the study population, some differences in mobility and physical fitness were observed. Possible selection to the most demanding fitness tests likely weakened the observed associations. However, we still found significant associations between estimated VO2peak test and work ability.

In addition to physical fitness, future examinations should take into account other explanatory factors, such as work conditions and work tasks, which we were not able to study in detail in the current study due to a small sample size and study population including mainly non-manual workers. It can be speculated that upper body and trunk strength are important aspects of physical fitness in occupations including heavy lifting and carrying tasks, whereas function of lower-extremities more likely plays a role in occupations including walking, squatting or kneeling. Therefore, the associations should be examined in large study populations with versatile work tasks and conditions. Longitudinal studies are also warranted to examine how physical fitness associates with development of work ability over time. To understand causal effects and provide practical recommendations, intervention studies examining the effect of physical fitness training on work ability among older adults are needed.

### Conclusions

Cardiorespiratory fitness, upper body strength and lower extremity functioning were positively associated with work ability among aging public sector workers. These findings suggest that although work life has probably changed to less strenuous, promoting aging workers’ physical fitness during the last years of their work careers could be one of the possible strategies to support aging workers’ work ability.

## Supplementary Information

Below is the link to the electronic supplementary material.

Supplementary file 1 (DOCX 22 kb)

## Data Availability

Anonymized partial datasets of the FIREA study are available by application with bona fide researchers with an established scientific record and bona fide organizations.
