# Supplementary material for: Work ability and physical fitness among aging workers: the Finnish Retirement and Aging Study
Source: Eur J Ageing. 2022 Jun 22;19(4):1301–10. doi: 10.1007/s10433-022-00714-1 (PMC9729519; doi:10.1007/s10433-022-00714-1)
Supplement: Supplementary file 1 — (DOCX 22 kb) [file 10433_2022_714_MOESM1_ESM.docx]

**Supplementary Table 1.** Interaction between physical fitness indicators and occupational status on work ability.

| Physical fitness indicator | Interaction between physical fitness indicator and occupational status^A^ | | | |
| --- | --- | --- | --- | --- |
|  | β^B^ | 95% CI | | p value |
| Estimated VO2peak (ml/kg/min) | 0.16 | -0.22 | 0.55 | 0.40 |
| Modified push-up test (number of repetitions) | -0.04 | -0.40 | 0.33 | 0.85 |
| Hand grip strength (N) | -0.05 | -0.39 | 0.29 | 0.77 |
| Sit-up test (number of repetitions) | -0.10 | -0.45 | 0.25 | 0.57 |
| Chair rise test time (s) | 0.01 | -0.33 | 0.34 | 0.97 |
| Maximal walking speed (m/s) | -0.06 | -0.40 | 0.27 | 0.72 |

^A^ Adjusted for age and gender.

^B^ Two-level occupational status, where manual vs. non-manual.
